# An Efficient *Stevia rebaudiana* Transformation System and *In vitro* Enzyme Assays Reveal Novel Insights into UGT76G1 Function

**DOI:** 10.1038/s41598-020-60776-y

**Published:** 2020-02-28

**Authors:** Qian Wu, Christophe La Hovary, Han-Yi Chen, Xu Li, Hayde Eng, Veronica Vallejo, Rongda Qu, Ralph E. Dewey

**Affiliations:** 1 Department of Crop and Soil Sciences, North Carolina State University, Raleigh, NC 27695 USA; 2 Present Address: Elo Life Systems, 3054 East Cornwallis Road, Durham, NC 27709 USA; 3 Present Address: Johnson County Community College, 12345 College Blvd, Overland Park, KS 66210 USA; 4 Department of Plant and Microbial Biology, North Carolina State University, Raleigh, NC 27695 USA; 5 PepsiCo, 700 Anderson Hill Rd, Purchase, NY 10577 USA

**Keywords:** Biotechnology, Molecular biology, Plant sciences

## Abstract

*Stevia rebaudiana* (Bertoni) is one of a very few plant species that produce zero calorie, sweet compounds known as steviol glycosides (SG). SGs differ in their sweetness and organoleptic properties depending on the number and positioning of sugar groups on the core steviol backbone. There is great interest of modulating the SG profiles of the Stevia plant to enhance the flavor profile for a given application in the food and beverage industries. Here, we report a highly efficient Agrobacterium-mediated stable transformation system using axillary shoots as the initial explant. Using this system, we generated over 200 transgenic Stevia plants overexpressing a specific isoform of *UGT76G1*. By comparing the SG profiles among independent transgenic events, we demonstrated that altering *UGT76G1* expression can change the ratios of specific SG species. Furthermore, using recombinant proteins produced in *E*. *coli*, we show that two closely related UGT76G1 isoforms differ in their substrate specificities, providing new insights into mechanisms underlying the diversity of SG profiles that are observed across Stevia germplasm. Finally, we found evidence suggesting that alternative and/or aberrant splicing may serve to influence the ability of the plant to produce functional *UGT76G1* transcripts, and possibly produce enzyme variants within the plant.

## Introduction

*Stevia rebaudiana* (Bertoni) is a self-incompatible plant species, and one of a few species in the *Stevia* genus whose leaves produce and accumulate high quantities of sweet steviol glycoside (SG) compounds^1^. SGs exist as a complex mixture of related compounds, with certain SG species conferring favorable sweetness characteristics while others are associated with a bitter or metallic taste^2^. As a natural sweetener that cannot be further metabolized in the human digestive tract, Stevia and the SGs have been rapidly gaining popularity in the food and beverage industries. The abundance and ratios of the SGs vary greatly among Stevia cultigens. Even within the same Stevia cultigen, the concentration and diversity of the SGs can vary in response to environmental factors such as light, water and nutrient availability^1,3–5^.

**Figure 1:**
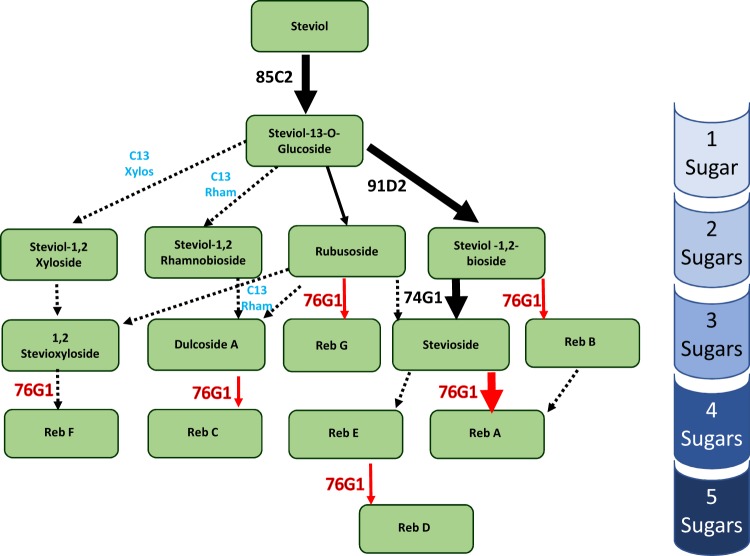
Simplified representation of the SG pathway. The largest arrows show the primary route of synthesis for the predominant SGs stevioside and Reb A. Steps of the pathway catalyzed by a UGT for which experimental evidence has been published are indicated by solid arrows. Steps of the pathway predicted to occur, but not experimentally demonstrated to be catalyzed by a specific UGT are indicated with dashed arrows. The UGT enzyme responsible, or proposed to be responsible, for a given reaction is indicated on the left of the arrow; steps where a non-glucose sugar is added are represented in blue type. The 76G1 enzyme that is the subject of this report is shown in red type, and steps shown to be associated with 76G1 herein (by *in vitro* and/or *in vivo* evidence) are indicated with red arrows.

The biosynthesis of SGs in Stevia has been extensively investigated and reviewed^2,6^. Figure 1 shows a simplified version of the pathway based on our current understanding. Most of the key reactions of SG biosynthesis are catalyzed by cytosolic UDP-dependent glycosyltransferases (UGTs). In the first characterization of the genes encoding UGTs of the SG biosynthetic pathway, UGT85C2, UGT74G1 and UGT76G1 were identified as being central to the production of the SGs^7^. Using *E*. *coli* extracts containing recombinant UGT proteins, it was shown that UGT85C2 functioned at the beginning of the pathway to catalyze the synthesis of steviol-13-O-glucoside from steviol. UGT74G1 displayed a broader substrate specificity, catalyzing the formation of steviol-monoside (presumably 19-O-glucoside) from steviol, a steviolbioside (presumably rubusoside) from steviol-13-O-glucoside, and stevioside from steviol-1,2-bioside. Recombinant UGT76G1 was also shown to be capable of recognizing multiple substrates. In addition to catalyzing the formation of RebA from stevioside, this enzyme was reported to glucosylate steviol-1,2-bioside to an “unknown” compound^7^.

More recent studies have yielded additional insights with respect to role of UGT76G1 in the production of SGs. Using *in vitro* assays of *Saccharomyces cerevisiae*-derived lysates, it was demonstrated that in addition to its well documented role in catalyzing the glucosylation of stevioside to produce Reb A, UGT76G1 was also capable of transferring glucose groups to position 3′ of the C13 glucose of steviol-1,2-bioside, rubusoside and Reb E to produce Reb B, Reb G, and Reb D, respectively^8^. This study also showed UGT76G1-mediated transfer of glucose groups to the 3′ position of the C19 glucose of various SG species, but at a much reduced efficiency in comparison to substrates with an available hydroxyl group on C3′ of the C13 glucose. All of the aforementioned SG species contain only glucose sugars attached to the core steviol backbone. The concept that UGT76G1 can also recognize SG species with alternative sugar groups was recently demonstrated through the use of *in vitro* assays in a study that showed UGT76G1 to be capable of catalyzing the synthesis of Reb C from dulcoside A, an SG species with a rhamnose moiety at C2′ of the C13 sugar^9^. The promiscuous nature of UGT76G1 enzymes appears to extend beyond metabolites of the SG pathway, as it has been recently shown that this enzyme can accept an array of alcohols, polyphenols, substituted monophenols, and glycosides as substrates when assayed *in vitro*^10^. Finally, an enzyme designated UGT91D2 has been shown to catalyze the synthesis of steviol-1,2-bioside from steviol-13-O-glucoside^11^.

Since the original characterization of UGTs of the steviol glycoside pathway^7^, the number of SG species known to exist within the Stevia plant has expanded greatly, particularly as analytical technologies have enabled the detection of species found in relatively minor abundance. Knowledge of the structures of the minor SG species, combined with our current understanding of the substrate specificities of the existing characterized UGTs of the SG pathway enables one to predict the route of synthesis of these minor SGs. Steps of the SG pathway where the enzyme responsible has been predicted, but not experimentally validated, are shown in Fig. 1 as dotted lines.

An examination of the UGT76G1-like entries found in GenBank suggests that at least three distinct isoforms exist within the Stevia germplasm. The original *UGT76G1* cDNA characterized corresponds to GenBank entry AY345974^7^ (which will be referred to as *76G1-AY* in this report). The predicted protein product of GenBank entry GQ259127 (*76G1-GQ*) displays 29 amino acid polymorphisms compared with 76G1-AY, with an overall shared amino acid identity of 94%. Although annotated in GenBank as encoding an enzyme called 76G2 rather than 76G1, the genomic sequence designated FJ607329 (possessing a 68 bp intron) predicts a protein product that is 93% identical to 76G1-AY (34 amino acid polymorphisms) and 96% identical to 76G1-GQ (19 amino acid polymorphisms). Another genomic GenBank entry (KC631816) is predicted to encode an enzyme that differs from 76G1-AY at one amino acid position, and thus likely represents an allelic variant of the same locus, rather than a distinct UGT76G1 paralog. Although it is not entirely clear how many distinct isoforms of *UGT76G1* exist within a single genome, a recent molecular and genetic study suggests that any given Stevia genome possesses a single *UGT76G1* locus, and that this locus is frequently heterozygous for alternative isoforms^12^. Whether these different isoforms display unique substrate specificities is unknown. In addition, understanding the degree by which enzymatic activities measured *in vitro* recapitulate *in vivo* activity within the Stevia plant also warrants further investigation.

To elucidate the specific function of UGTs in the SG biosynthetic pathway in Stevia, the alteration of gene activity via transgene manipulation is the most straightforward and reliable strategy. The manipulation of gene expression *in vivo*, however, requires an efficient Stevia tissue culture and transformation system. There are several reports describing the propagation of Stevia tissue cultures initiating from different explants, and a diverse array of tissue culture conditions have been reported^13–22^. Until very recently, however, there are minimal reports in Stevia involving genetic transformation. In one study, genes of the SG biosynthetic pathway were targeted using RNAi constructs in a transient expression system^23^. In another report, a single stable transformation event of the *GUS* reporter gene was described using Agrobacterium-mediated transformation of leaf discs^24^. Furthermore, successful recovery of transgenic Stevia callus, but not whole plants, was documented using a *GUS-bar* construct delivered via particle bombardment^25^. Very recently, a research group from the University of Singapore described the stable transformation of Stevia using kanamycin as the selectable marker and GFP as a reporter gene^9,26^.

In this paper, we report an efficient Agrobacterium-mediated stable transformation system for Stevia based on hygromycin B (Hyg) as the selection agent. Using this system, we generated over 200 stably transformed Stevia plants overexpressing the gene encoding 76G1-AY. In addition, using an *E*. *coli*-based recombinant expression assay we demonstrated that 76G1-AY and 76G1-GQ differ in their substrate specificities, with the former enzyme recognizing a broader range of substrates than the latter. Finally, sequence analysis of populations of *UGT76G1* cDNAs revealed that an array of splice variants exists within at least two Stevia cultigens, providing yet another likely form of *UGT76G1* regulation. Collectively, the results presented here advance our ability to investigate gene function in Stevia through the development of an efficient transformation system, as well as provide new insights with respect to *UGT76G1* function and its role toward producing the various metabolites and products of the SG biosynthetic pathway.

## Results

### Stevia clonal propagation

As a first step in establishing a Stevia transformation protocol, we surveyed a collection of cultigens for their relative responsiveness to tissue culturing. Twenty-seven different Stevia cultigens were clonally propagated by taking either the young shoot apex or nodal cuttings (a node flanked by sections of internode on both sides) from greenhouse-grown Stevia plants. Clonal cultures were easily initiated and maintained with a simple ½ MS medium (Table 1). After establishment in tissue culture medium, root formation was typically observed about 5–7 days after each subsequent sub-culture.

**Table 1:** Media developed for Stevia tissue culture and transformation.

	MS	Sucrose (g/L)	MES (g/L)	pH	Agar (g/L)	2,4-D* (mg/L)	BAP* (mg/L)	GA* (mg/L)	NAA* (mg/L)	Gln* (mg/L)	CA* (mg/L)	Hyg* (mg/L)	Ticar* (g/L)	As* (µM)
½ MS	0.5X	15	0.5	5.7–5.8	6	—	—	—	—	—	—	—	—	—
ASI	0.5X	15	0.5	5.7–5.8	6	—	1	—	—	—	—	—	—	—
AgI	1X	30	—	5.5–5.7	—	—	—	—	—	—	—	—	—	100
CC	1X	30	0.5	5.7–5.8	6	1	0.5	1	—	50	100	—	—	100
CI	1X	30	0.5	5.7–5.8	6	1	0.5	1	—	50	100	6	0.52	—
DM	1X	30	0.5	5.7–5.8	6	—	2	—	0.1	50	100	4	0.52	—
RMH	0.5X	15	0.5	5.7–5.8	6	—	—	—	—	—	—	4	0.52	—

1/2 MS: basic clonal propagation and culture maintenance medium; ASI: Axillary Shoot Induction medium; AgI: Agrobacterium Inoculation medium; CC: Agrobacterium-explant Co-Culture medium for pale green axillary shoots; CI: Callus Induction and selection medium; DM: Callus Differentiation and selection medium; RMH: Rooting and Hyg selection medium. Reagents with an asterisk were added after autoclaving and cooling down to 55 °C. The pH of all media was adjusted before sterilization by autoclaving.

Abbreviations and vendors.

MS: Murashige and Skoog medium (Sigma-Aldrich, St. Louis, MO); Sucrose (Caisson, Smithfield, UT); MES (Fisher Scientific, USA); Agar (Sigma-Aldrich, St. Louis, MO); 2,4-D: 2,4-Dichlorophenoxyacetic acid (Sigma-Aldrich, St. Louis, MO); BAP: 6-Benzylamino purine (Sigma-Aldrich, St. Louis, MO); GA: Gibberellic Acid (Caisson, Smithfield, UT); NAA: Α-Naphthaleneacetic acid (Sigma-Aldrich, St. Louis, MO); CA: Casamino Acids (Fisher Scientific, USA); Gln: L-Glutamine (Sigma-Aldrich, St. Louis, MO); As: 4′-Hydroxy-3′,5′-dimethoxyacetophenone, Acetosyringone (Sigma-Aldrich, St. Louis, MO); Ticar: Ticarcillin/Clavulanate (15/1) (Gold Biotechnology, St. Louis, MO); Hyg: Hygromycin B (Gold Biotechnology, St. Louis MO).

Out of the 27 different cultigens tested, NTV1 responded especially well to the callus induction medium, and callus regeneration to shoots occurred in a relatively short time period (4–6 weeks) and at high efficiency (i.e. the number of successful regeneration events per callus). Furthermore, NTV1 displays a relatively balanced steviol glycoside profile (Supplementary Table 2), which is desirable for experiments designed to test the effects of SG pathway gene manipulation in influencing the flux of metabolites through the pathway. Therefore, we selected NTV1 as the cultigen to be used for transformation.

### Stevia transformation and molecular verification

**Figure 2:**
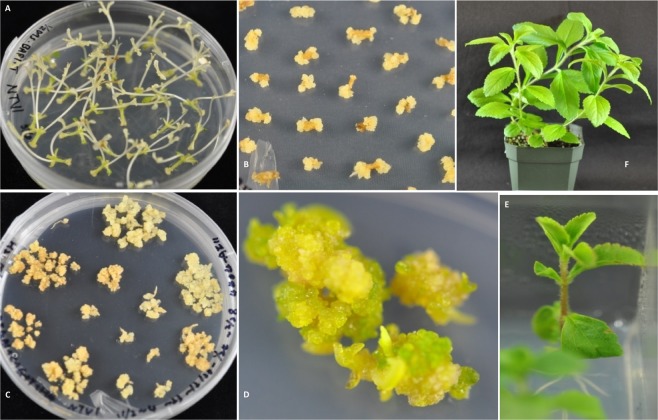
Agrobacterium-mediated transformation using the axillary shoot as the explant. (**A**) Axillary shoots induced from nodal segments in the dark; (**B**) callus induced from segments of axillary shoots; (**C**) callus grown on Hyg selection medium; (**D**) shoot differentiation on Hyg selection medium; (**E**) regenerated shoot rooted on Hyg selection medium; and (**F**) transgenic Stevia plants established in soil.

**Figure 3:**
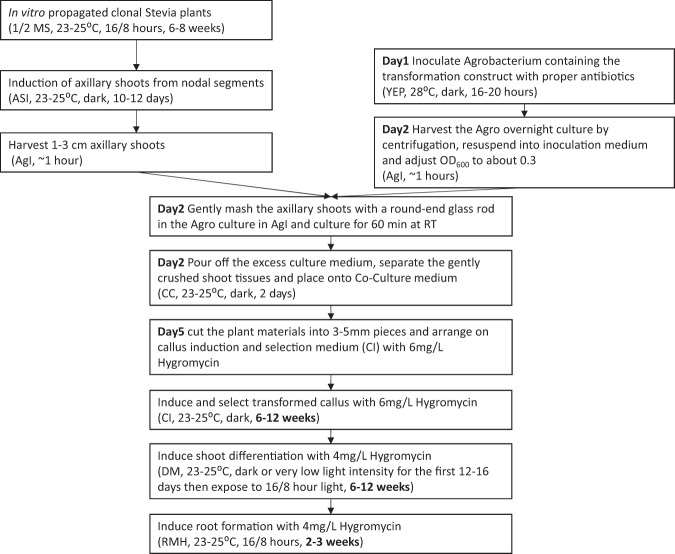
Flow chart of the Stevia transformation procedure. ASI, AgI, CC, CI, DM or RMH are the medium used at each step (see details in Table 1).

The transformation protocol developed in this study is presented in detail in Materials and Methods, and is summarized in Figs. 2 and 3. What we consider to be among the most critical features for achieving efficient Stevia transformation are the following: (1) development of a callus induction system using young axillary shoots, as this represents a young, less differentiated source of explant; (2) initiating transformation and callus induction simultaneously, as this shortens the transformation timeline and reduces the risk of somaclonal variation that could occur as a result of extended culture time; and (3) gently mashing the axillary shoots with a round-end glass rod, which greatly increases callus induction efficiency and significantly increases the interaction between explant and Agrobacterium.

**Figure 4:**
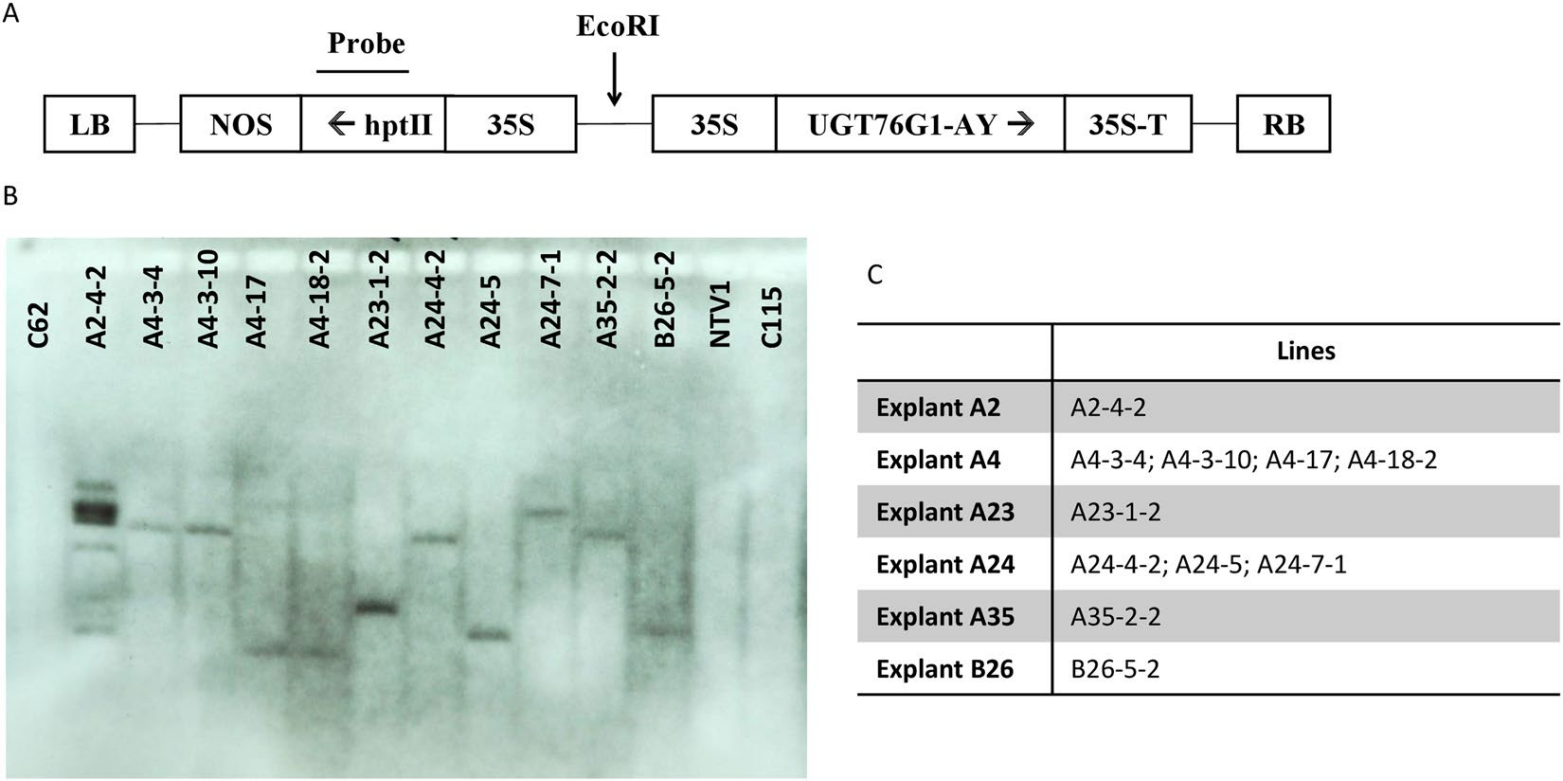
Southern blot detection of selected transgenic Stevia plants. (**A**) T-DNA section of UGT76G1 overexpression construct. RB: Right Border; LB: Left border; UGT76G1-AY: *Stevia rebaudiana* UDP-glycosyltransferase 76G1 (NCBI AY345974); 35S: CaMV 35S promoter; 35S-T: CaMV 35S terminator; NOS: nopaline synthase terminator. hptII: *hygromycin phosphotransferase*, plant selection marker gene. EcoRI was used for genomic DNA digestion and the probe used for the Southern blot was specific to the hptII sequence. (**B**) DIG-labeled Southern blot analysis of 11 selected transgenic lines (A and B lines) and non-transgenic controls (C62, NTV1 and C115). (**C**) Summary of the explant source and transgenic line relationship.

We utilized the optimized transformation protocol to transform cultigen NTV1 with construct 35S:UGT76G1-AY (Fig. 4A). Using this protocol we were able to obtain a little more than 200 transgenic plants, all of which originated from 6 independent explants out of approximately 200 starting explants. Therefore, the efficiency of transformation would be calculated as 3% if efficiency is defined as the number of explants that produce at least one transgenic event over the total number of explants that were transformed (×100%). All plants were rooted in a medium containing 4 mg/L Hyg; rooting was not observed in any non-transgenic control shoot transferred to this medium. To further confirm that the Hyg resistant plants contained the transgene, we conducted PCR assays using primers designed to amplify the junction of the 35S promoter and 76G1-AY within the 35S:UGT-76G1-AY-hptII construct, using genomic DNA extracted from each of the <200 putative transgenic plants. The predicted transgene-specific amplification product was detected in all transgenic plants (data not shown). This result suggests that hygromycin selection is very efficient for Stevia, precluding the need for a reporter gene such as GFP to optimize the selection process.

Eleven transgenic plants were selected for Southern blot analysis. Each of the six originating explants was represented by 1–4 plants in this experiment, as shown in Fig. 4C. The presence of the transgene was tested using the *hptII* selectable marker gene as the hybridization probe. Positive signals were detected from all 11 PCR positive plants, and no hybridization was observed using genomic DNA from control plants. Based on the similarities in the banding pattern of the Southern blot, of the four plants originating from Explant A4, plants A4-3-4 and A4-3-10 could be clonal, and plants A4-17 and A4-18-2 could be clonal as well. Of the three plants originating from Explant A24, all three clearly represent independent transformation events. The complex banding pattern of event A2-4-2 showed multiple copies of the transgene incorporated into the genome. All other plants displayed a simple pattern, with the transgene likely present as a single copy.

In both cases where multiple plants were examined that were derived from independent shoots arising from the same original explant (Explants A4 and A24), unique transgene integration events were observed. Because of this phenomenon, it can be reasonably assumed that the number of unique T_0_ events generated in this experiment is considerably greater than the nine events documented in Fig. 4. Calculating overall transformation efficiency in terms of unique T_0_ events recovered becomes more complicated, and would require analyzing numerous individuals originating from each transformation-positive explant by Southern blotting. Although we don’t have a good estimate of transformation efficiency when calculated in terms of the number of unique T_0_ events recovered per total number of explants inoculated, it was clearly much higher than the 3% calculated above, when efficiency was defined simply in terms of the percentage of explants yielding at least one positive transformation event.

Since the transgenic plants generated in this study were designed to overexpress *76G1-AY* under the control of the constitutive CaMV 35S promoter, real time quantitative PCR (RT-qPCR) was conducted to measure *UGT76G1* expression levels. Due to the high similarity among the *UGT76G1* cDNA sequences reported in GenBank, the primers were designed against sequences conserved across all four NCBI annotated *UGT76G1*variants. Our results showed a wide range of *UGT76G1* expression levels, with most of the transgenic lines showing an increase in transcript accumulation when compared to endogenous expression levels in non-transgenic controls (Supplementary Fig. 1).

### Steviol glycoside profile analysis

To evaluate the SG profiles of the transgenic lines, leaves from 6–12 clonally propagated plantlets of each line were pooled and subjected to HPLC-MS analysis. Each of the 11 lines that had been analyzed by Southern blotting and RT-qPCR for *UGT76G1* expression were included. As controls, three non-transgenic lines were used that had been sub-cultured and maintained simultaneously and within the same growth chambers. A particular emphasis was placed on those compounds that according to our understanding of the SG pathway had the potential of serving as the substrate or product of a UGT76G1-catalyzed reaction (see Fig. 1) and could be detected in high enough abundance to be reliably interpreted. These compounds included: steviol-1,2-bioside, rebaudioside B (Reb B), stevioside, rebaudioside A (Reb A), rebaudioside E (Reb E), rebaudioside D (Reb D), dulcoside A, rebaudioside C (Reb C), rubusoside and rebaudioside G (Reb G). In addition to simply measuring the abundance of each SG compound, we calculated the ratios between each potential substrate/product pair, as a reduction in this ratio would be suggestive of increased UGT76G1 activity in the overexpression lines.

The results of the SG analyses are shown in Table 2. The glycoside profiles among the three non-transgenic control lines was very consistent; in contrast, the transgenic lines showed considerable variability. Most studies of UGT76G1 have focused on its ability to convert stevioside to Reb A. In plants possessing 35S:76G1-AY constructs, there is a very good correlation between transgene expression and a reduction in the stevioside/Reb A ratios. In the three WT controls, stevioside accumulation ranged between 2.0 to 2.6-fold higher than Reb A. Five transgenic lines were predicted to accumulate *UGT76G1* transcripts by more than 5-fold of that observed in controls (lines TA4-17, TA4-18-2, TA24-7-1, TA35-2-2 and TB26-5-2; Supplemental Figs. 1 and 2). In four of these five lines stevioside and Reb A are in essentially equal in abundance, and in the fifth line (TA4-17) the amount of Reb A is twice that of stevioside. This decrease in putative substrate/product ratios also extends to the four other comparisons shown in Table 2 for the five highest expressing 35S:76G1-AY lines. These results strongly suggest that in addition to stevioside, the 76G1-AY enzyme is also capable of utilizing steviol-1,2-bioside, rubusoside, Reb E, and dulcoside A as substrates *in vivo*.

**Table 2:** SG profile analysis of transgenic Stevia plants.

Compound Name	Transgenic lines											Non transgenic lines		
	+	++	++	++++	+++	++	++	++	+++	+++	++++	+	+	+
	**TA2-4-2**	TA4**-**3**-**4	TA4**-**10	TA4**-**17	TA4**-**18**-**2	TA23**-**1**-**2	TA24**-**4**-**2	TA24-5	TA24-7-1	TA35-2-2	TB26-5-2	T62	T86	T115
Steviol-1,2-bioside	**0**.**10**	0.05	0.06	0.04	0.04	0.05	0.04	0.06	0.03	0.07	0.05	0.04	0.04	0.04
Reb B	**0**.**01**	0.06	0.06	0.09	0.06	0.08	0.05	0.05	0.06	0.16	0.09	0.04	0.04	0.04
Ratio 1	**7**.**60**	**0**.**88**	**0**.**88**	**0**.**39**	**0**.**64**	**0**.**61**	**0**.**86**	**1**.**15**	**0**.**52**	**0**.**42**	**0**.**53**	**0**.**94**	**0**.**98**	**0**.**96**
Stevioside	**17**.**70**	8.96	10.80	5.95	6.89	7.50	8.22	9.37	5.75	10.47	8.15	7.60	7.56	5.66
Reb A	**0**.**02**	4.97	4.98	12.19	6.99	5.40	4.28	3.41	5.61	10.20	7.63	3.82	3.05	2.17
Ratio 2	**791**.**76**	**1**.**80**	**2**.**17**	**0**.**49**	**0**.**99**	**1**.**39**	**1**.**92**	**2**.**75**	**1**.**03**	**1**.**03**	**1**.**07**	**1**.**99**	**2**.**48**	**2**.**61**
Reb E	**0**.**46**	0.15	0.18	0.05	0.12	0.09	0.10	0.13	0.06	0.10	0.07	0.13	0.14	0.14
Reb D	**0**.**00**	0.18	0.18	0.25	0.22	0.16	0.13	0.12	0.13	0.27	0.19	0.14	0.10	0.13
Ratio 3	**272**.**14**	**0**.**86**	**1**.**00**	**0**.**21**	**0**.**56**	**0**.**57**	**0**.**80**	**1**.**04**	**0**.**43**	**0**.**38**	**0**.**38**	**0**.**97**	**1**.**30**	**1**.**09**
Dulcoside A	**0**.**68**	0.25	0.33	0.11	0.21	0.19	0.24	0.34	0.14	0.22	0.16	0.21	0.21	0.28
Reb C	**0**.**03**	1.50	1.65	2.16	1.51	1.45	1.28	1.22	1.33	2.36	1.79	1.22	1.12	0.72
Ratio 4	**20**.**32**	**0**.**17**	**0**.**20**	**0**.**05**	**0**.**14**	**0**.**13**	**0**.**19**	**0**.**28**	**0**.**10**	**0**.**09**	**0**.**09**	**0**.**18**	**0**.**19**	**0**.**39**
Rubusoside	**0**.**14**	0.05	0.05	0.04	0.03	0.05	0.05	0.08	0.05	0.06	0.06	0.04	0.03	0.04
Reb G	**0**.**01**	0.10	0.10	0.25	0.11	0.13	0.09	0.08	0.11	0.25	0.18	0.06	0.06	0.07
Ratio 5	**10**.**80**	**0**.**50**	**0**.**44**	**0**.**16**	**0**.**30**	**0**.**36**	**0**.**57**	**1**.**01**	**0**.**41**	**0**.**25**	**0**.**34**	**0**.**66**	**0**.**48**	**0**.**63**

Leaf samples were collected from 6-12 vegetatively propagated tissue cultured plants of each line and pooled for analysis. The content is presented in µg/mg dry weight. Top row indicates UGT76G1 expression levels relative to wild type controls, as determined by RT-qPCR analysis. + = 1–2 fold; ++ = 2–5 fold; +++ = 5–10 fold; ++++ = >10 fold. Ratio 1 is steviol-1,2-bioside/Reb B; Ratio 2 is stevioside/Reb A; Ratio 3 is RebE/Reb D; Ratio 4 is dulcoside A/Reb C; Ratio 5 is rubusoside/Reb G.

The line with the most unusual SG profile was TA2-4-2, which is also the only transgenic line of the group that displayed a complex banding pattern in the Southern blotting assays, indicative of high transgene copy number (Fig. 4B). For TA2-4-2, the SG profile is heavily skewed in favor of the putative UGT76G1 substrates, with all five of the proposed Reb A, Reb B, Reb C, Reb D and Reb G products being found in negligible amounts in this line. Although the RT-qPCR results predicted the *UGT76G1* transcript levels in this line to be similar to the WT (Supplemental Fig. 1), the SG profile is strongly supportive of TA2-4-2 being a co-suppression line, a phenomenon whose occurrence is especially high in transgenic plants possessing multiple transgene insertions^27^. Overall, the transgenic plant data shown in Table 2 strongly supports the conclusion that 76G1-AY can utilize steviol-1,2-bioside, rubusoside, stevioside, Reb E and dulcoside A as substrates *in vivo*, and that alteration of *76G1-AY* expression levels can lead to substantial changes in the SG profile of the plant.

### Substrate specificities of UGT76G1 isoforms assayed *in vitro*

As an additional objective, we wanted to establish whether the closely related UGT76G1 isoforms, 76G1-AY and 76G1-GQ, were functionally equivalent. The original characterization of 76G1-AY showed that the enzyme was capable of the glucosylation of both stevioside and steviol-1,2-bioside^7^. This would suggest that the enzyme is capable of recognizing steviol-13-O-glucoside molecules that already have a sugar on position C2′ of the C13 glucose, and can attach an additional glucose at position C3′. According to this presumption, in addition to stevioside and steviol-1,2-bioside, the compounds Reb E and dulcoside A would also represent potential UGT76G1 substrates as shown in Fig. 1. The SG data from the 35S:76G1-AY transgenic plants also supports the hypothesis that this enzyme is capable of recognizing all four of these SG species as substrates.

**Figure 5:**
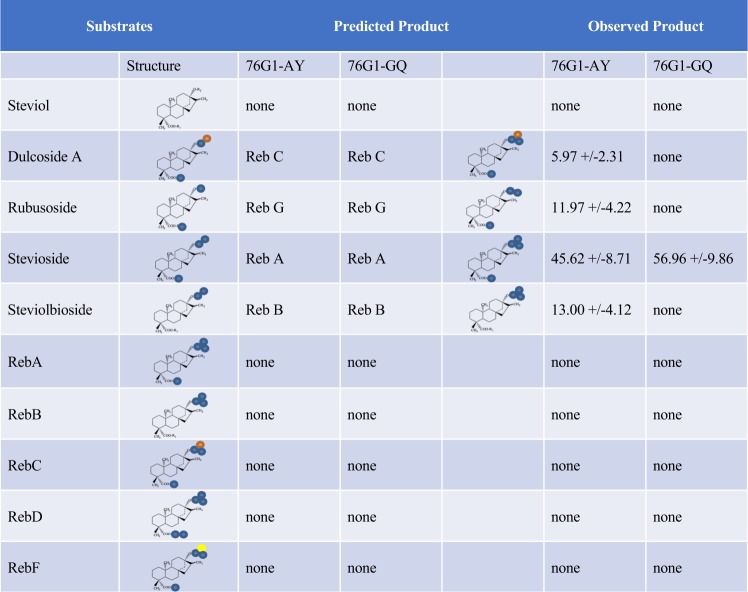
Metabolites of the SG pathway included in UGT76G1 *in vitro* assays. In reactions that yielded a product in the assays, the Km was calculated and is shown in the Observed Product column (+/− standard error). Structures are shown as the core steviol molecule decorated with one or more sugars. Blue circles represent glucose; orange circles represent rhamnose; the yellow circle represents xylose.

To directly test the substrate specificities of 76G1-AY and 76G1-GQ, the respective cDNAs were cloned into *E*. *coli* expression vector pET30 and assayed for glucosyltransferase activity in the presence of various SG metabolites *in vitro*. Although the constructs were engineered to place an N-terminal His-tag on the recombinant 76G1 proteins, we were only able to detect *in vitro* enzyme activity using crude *E*. *coli* lysates, and not from protein fractions that had been purified over a Nickel column. This same phenomenon was also observed by Richman *et al*.^7^ who therefore conducted all of their *in vitro* studies using crude lysates. To compare the substrate specificities of 76G1-AY and 76G1-GQ, *in vitro* assays were conducted using all of the SG compounds commercially available from Chromadex (steviol, dulcoside A, rubusoside, stevioside, steviol-1,2-bioside, Reb A, Reb B, Reb C, Reb D and Reb F). No glycosylation products were observed in *E*. *coli* lysates containing the empty vector control plasmid. Assay results from lysates containing 76G1-AY or 76G1-GQ are shown in Fig. 5.

Similar to its original characterization^7^, we observed that 76G1-AY was capable of recognizing both stevioside and steviol-1,2-bioside as substrates. Furthermore, reaction products were also observed when two additional SG compounds were included in the assays (compounds not tested in the prior study): dulcoside A and rubusoside. For dulcoside A, this result is not surprising, as the addition of a glucose molecule at position C3′ would lead to the synthesis of Reb C. The glucosylation of rubusoside, however, was not as obvious, as it is contrary to the presumption that a sugar must occupy C2′ of the C13 glucose in order to serve as a substrate for a UGT76G1 enzyme. Should 76G1-AY be capable of glucosylating position C3′ of rubusoside in the absence of a sugar at C2′, the product would be rebaudioside G (Reb G), an SG species that has only recently be shown to be among the SG repertoire found in Stevia. The exact nature of the SG species produced by 76G1-AY-mediated glucosylation of rubusoside in our assays, however, remains to be validated.

Despite sharing 94% amino acid sequence identity, 76G1-AY and 76G1-GQ were not completely redundant according to the results of the *in vitro* assays. 76G1-GQ displayed a much narrower substrate specificity than 76G1-AY, as only stevioside was recognized as a substrate by this UGT76G1 isoform (Fig. 5).

### UGT76G1 transcript diversity

**Figure 6:**
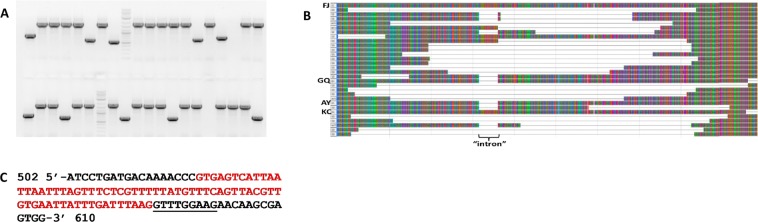
Transcript diversity in Stevia. (**A**) Gel electrophoresis of PCR amplification products from randomly cloned *UGT76G1* cDNAs from cultigen NTV1. In addition to full length copies of *UGT76G1* cDNAs, shorter products were also observed at a high frequency. (**B**) Alignment of various aberrant cDNA products with wild type cDNA, along with genomic sequences found in GenBank (FJ = FJ607329; GQ = GQ259127; AY = AY345974; KC = KC631816). Each row indicates a unique cDNA sequence, and the location of the intron is noted. Image was generated using Mega 5.0 software (www.megasoftware.net) (**C**) Localized region of *UGT76G1* (specifically KC631816) containing the 68 bp intron (in red type); numbering is relative to the ATG start codon. In some cDNA species, alternative splicing led to the elimination of the nine bp adjacent to the normal 3′ splice site (underlined).

In the course of isolating full length UGT76G1 cDNAs, first for generating the 35S:76G1-AY overexpression construct for the transformation experiments, and later for expressing both 76G1-AY and 76G1-GQ in the *E*. *coli-*based *in vitro* system, we observed a particularly broad array of sequence variants. Specifically, a large percentage of presumed splice variants of the *UGT76G1* transcripts were observed. To better understand the nature and relative frequencies of these transcript variations, we isolated total RNA from the leaves of two distinct Stevia cultigens (NTV1 and SDSV32), and amplified the region encompassing the *UGT76G1* ORFs using primers that had been designed to recognize both the *76G1-AY* and *76G1-GQ* isoforms. The amplification products were cloned into a TA vector and analyzed by gel electrophoresis and/or DNA sequencing. In total, 82 and 107 *UGT76G1* cDNA sequences were obtained and characterized from cultigens NTV1 and SDSV32, respectively. A surprising degree of *UGT76G1* cDNA diversity was observed in both the NTV1 and SDSV32 backgrounds (Fig. 6). The majority of the cDNA variations appeared to be the consequence of imperfect splicing at the single intron found with the *UGT76G1* genes, or through cryptic splicing events unrelated to the intron. Each of these phenomena was observed in multiple independent cDNA sequences. The fact that atypical splicing events were observed across independent amplification experiments and in separate Stevia cultigens suggests that these were not simply PCR or cloning artifacts.

As shown in Table 3, 70% and 55% of the total cDNA sequences characterized from NTV1 and SDSV32, respectively, corresponded to WT *76G1-*AY or *76G1-*GQ (in the latter case a presumably cultigen-specific single nucleotide polymorphism was observed that differs from the sequence in GenBank but doesn’t alter the amino acid sequence). 11% and 22% of the total cDNAs sequenced from NTV1 and SDSV32, respectively, retained the sequences corresponding to the 68 bp intron. Control reactions lacking reverse transcriptase failed to yield amplification products, which strongly suggests that these products are not the result of genomic DNA contamination. The majority of the remaining cDNA sequences were substantially shorter in length, and sequence analysis showed them to be interior truncations of varying sizes (Fig. 6A,B), ranging from 9 bp to 1.2 kb. We observed a similar phenomenon in each of the two Stevia cultigens examined, though a higher percent of the cDNAs from SDSV32 (45%) were abnormal compared to NTV1 (30%).

**Table 3:** Frequencies of transcript variants observed in *UGT76G1* cDNAs from Stevia cultigens NTV1 and SDSV32.

	NTV1 No. (%)	SDSV32 No. (%)
Unspliced intron	11 (13%)	22 (21%)
Abherrant internal truncations	14 (17%)	26 (24%)
Wild type 76G1-AY or 76G1-GQ	57 (70%)	59 (55%)
No. of total sequences	82	107

One of the more interesting splicing variants involved an extension of the intron by an additional nine bp at the 3′ splice junction (Fig. 6C). This variant was found independently three times. The vast majority of plant introns are bordered by the dinucleotide GT at the 5′ end and AG at the 3′. From close examination of the sequence, the simplest explanation is that the spliceosome complex occasionally bypasses the AG at the 3′ splice junction and instead recognizes the next AG encountered, which is nine nucleotides downstream, leading to the creation of the observed 9 bp deletion variant. Proteins produced from this alternatively spliced transcript would differ from the wild type in that the Arg at position 174 would be converted to a Gln residue, and the adjacent Leu-Glu-Glu amino acids normally found at positions 175–177 would be missing.

## Discussion

Numerous studies have been published relating to the tissue culturing of Stevia, and a diverse array of culture conditions and medium compositions have been described. The impetus for much of the tissue culturing studies in Stevia has been due to the fact that as a self-incompatible, obligate out-crossing species, the only way to maintain genetic uniformity of a desired cultigen is through vegetative clonal propagation. With respect to genetic transformation, working with self-incompatible plant species limits the use of certain explants that work well for transformation in other plant species, such as immature embryos, seeds, and young seedlings, due to the lack of genetic uniformity of these in Stevia. Ensuring genetic uniformity is particularly important when the goal involves modification of the SG content, given the tremendous variation observed in the SG profiles observed across different Stevia germplasm^2,12^.

In order to select the specific Stevia background that would likely be favorable for transformation, we subjected 27 diverse cultigens to a variety of culturing conditions. We found the general conditions for the maintenance and/or multiplication of shoots to be straight forward, with no special requirement for plant growth hormones needed among the 27 cultigens when using either the shoot apex or nodal segments as the explant for propagation (data not shown). Substantial differences did occur, however, in the number of shoots obtained per explant, and the speed with which the new shoots would grow. These experiments led to the selection of NVT1 as the cultigen determined to be most responsive to culturing, and thus the background of choice for transformation.

We established a system using young axillary shoots cultured in the dark as the explant to carry out Agrobacterium-mediated transformation. We considered young axillary shoots as an explant of choice due their potential for possessing a high percentage of young, not fully differentiated cells. Subsequent induction of callus and the maintenance of callus under Hyg selection was also conducted in the dark until regeneration. Very recently, researchers reported using tissue cultured leaves as the explant and successfully obtained stable transgenic Stevia plants^9,26^; this suggests that both types of originating explant are suitable for efficient transformation. Similar to our results, this same research group also concluded that a prolonged period of dark incubation was critical for obtaining high efficiency Stevia transformation. In many plant species, culturing callus tissue in the dark is required for optimal regeneration. In the case of Stevia, dark growth conditions also limit the production/accumulation of SGs. Although it is not clear whether SGs have a role in plant-bacteria interactions, it is possible that keeping SGs at a minimum concentration may be a factor in realizing high transformation efficiency.

In our transformation system, Hyg selection was the only means needed for selecting transgenic events. Our results showed that regenerated Stevia plants rooted in a medium containing 4 mg/L Hyg were 100% transgenic. Prior to using the *hptII* gene as a selectable maker, we made several attempts using vectors contain *nptII* to confer kanamycin resistance. When using kanamycin resistance for selection, however, we consistently observed nontransgenic escapes that were able to form shoots (data not shown). The problem of being able to find a level of kanamycin that could completely inhibit regeneration of non-transgenic Stevia, while enabling adequate overall regeneration rates was also encountered by Zheng *et al*.^26^. To overcome this obstacle, a GFP reporter construct was simultaneously introduced by that group and transgenic plants were selected through a combination of antibiotic pressure and visual selection for GFP using fluorescence microscopy. Our results suggest that if one uses *hptII* as the selectable marker gene, antibiotic selection alone is sufficient for enabling Stevia transformation without the use on additional reporter gene for visual selection. Overall, the transformation system described here should broaden the scope of Stevia transformation by showing the successful use of axillary shoots as the explant and describing conditions whereby the *hptII* gene can be used as the sole means of selection in obtaining high efficiency Stevia transformation.

Despite being the subject of several prior research studies, questions still remain regarding the role of *UGT76G1* in determining the ultimate SG profile in Stevia, as well as the degree to which the SG profile may be altered through modification of its expression. Using the Agrobacterium-mediated transformation protocol described above, we obtained several independent stable transgenic Stevia lines overexpressing a specific *UGT76G1* isoform (76G1-AY). The transgenic lines showed varying levels of *UGT76G1* expression, as well as differing patterns of SG accumulation in comparison to the non-transgenic control lines. Especially when comparing the ratios of individual SG compounds in the transgenic lines expressing *76G1-AY* at least five-fold higher than the controls (TA4-17, TA4-18-2, TA24-7-1, TA35-2-2 and TB26-5-2), a very consistent trend was observed between the ratios of the predicted 76G1-AY substrates and their presumed products. In the three independent control lines, stevioside was the predominant SG, accumulating to levels 2.0–2.5 fold higher than the next most abundant SG, Reb A (Table 2). In four of the five high *76G1-AY* expressing lines the ratio is very close to 1:1, while in the other line (TA4-17) the ratio is 2:1 in favor of Reb A. A skewing of the dulcoside A:Reb C ratio is also evident, though not as dramatic given that Reb C content is normally much greater than dulcoside A in control plants. The effects of *76G1-AY* overexpression on the stevioside:Reb A, and dulcoside A:Reb C ratios in this study are very consistent with similar transgenic plant results recently reported^9^. These four SG compounds were the only ones analyzed in that study, however, due to limitations in their detection of the less abundant SG species. In our study, we were able to extend the alteration in putative substrate/product relationships to steviol-1,2-bioside:Reb B, Reb E:Reb D, and rubusoside:Reb G as well. In each case, the ratios were strongly shifted in favor of the putative product in the five highest *76G1-AY* overexpressing lines. Of the six steps of the pathway shown in Fig. 1 for which UGT76G1 has been proposed to be involved, the stevioxyloside:Reb F is the only relationship that wasn’t validated *in vivo* in this study, due to our lack of suitable standards for these SG compounds.

Line TA2-4-2 was exceptional in that it was the only transgenic line that displayed a complex transgene insertion pattern indicative of carrying several copies of the transgene (five or more), a phenomenon frequently associated with co-suppression phenotypes. Although our RT-qPCR results did not show a clear distinction from WT, the unique SG pattern strongly supports the interpretation that both the transgene and endogenous copies of *UGT76G1* are silenced in this line. In line TA2-4-2, for all five of the potential substrate:product relationships indicated in Fig. 1 that we were able to measure, the ratio is dramatically skewed in favor of the substrate; all five products are present in negligible quantities. Overall, the results of the *76G1-AY* overexpression lines and the TA2-4-2 co-suppression line strongly support the model of Fig. 1 that postulates UGT76G1 as being the predominant, and likely sole, enzyme responsible for the production of Reb A, Reb B, Reb C, Reb D and Reb G *in vivo*.

To further investigate and confirm the function of UGT76G1, we conducted *in vitro* assays using recombinant 76G1-AY and 76G1-GQ proteins with UDP-glucose as the sugar donor and each SG species available from Chromadex as substrates. For 76G1-AY, the *in vitro* results were in perfect alignment with the results obtained *in vivo* for plants transformed with the 35S:76G1-AY transgene, as dulcoside A, rubusoside, steviol-1,2-bioside and steviolbioside were all utilized as substrates (Reb E was not tested as it was not available from Chromadex). These results are consistent with those reported using recombinant enzyme produced in yeast with respect to glucosylation of the glucose moiety attached at position C13, but differed in that we detected no activity when Reb A or Reb D were presented as substrates, whereas Reb I and Reb M were detected in the aforementioned study, indicative of glycosylation at the C19 glucose^8^. Given that the efficiencies of catalyzing 1,3-glucosylations at the C13-attached glucose were reported to be far greater than those at the glucose on C19 in the prior study^8^, combined with their use of far greater incubation times (18 hr versus 2 hr), it is likely that our assays were not sensitive enough to detect glucosylations positioned at the C19 glucose.

Although our *in vitro* and *in vivo* results coincided very well with respect to 76G1-AY, notable differences were apparent when the activity of 76G1-GQ was measured *in vitro*. Unlike 76G1-AY which displayed the predicted broad substrate specificity across several SG species, we could only detect *in vitro* enzymatic activity for 76G1-GQ using stevioside as the substrate. This suggests that the amino acid polymorphisms that exist between these two closely related isoforms alter the breadth of the enzyme’s substrate specificity. Previous studies involving *UGT76G1* have largely focused on the *76G1-AY* isoform^7–9^. In the one study that investigated the *76G1-GQ* variant (referred to therein as UGTSr), the authors were unable to recover activity from *E*. *coli* extracts, and instead relied on *in vitro* assays using extracts from tobacco suspension cultures expressing *76G1-GQ*^28^. In that study, however, stevioside was the only SG substrate tested.

We observed a surprising amount of size diversity when *UGT76G1* cDNAs were isolated and sequenced from Stevia leaves, an observation that was consistent across two distinct cultigens. The great majority of the atypical cDNAs would be predicted to encode nonfunctional products, as they involved the failure to splice out the intron (which would give rise to a frame-shift), large internal truncations, or both (Fig. 6). The alternative splicing and/or truncated transcript variants characterized were all internal with respect to a wild type transcript. This is likely a consequence of the approach used where the cDNA sequences described in this study were recovered using primers flanking the full length *UGT76G1* ORF. Therefore, potential transcript variants that may have lacked an intact 5′ or 3′ end would not have been amplified, so the total percentage of nonfunctional transcripts may even be greater than the 30% and 45% estimated for NTV1 and SDSV32, respectively (Table 3). Very recently, *UGT76G1* transcripts retaining the 68 nt intron sequence were shown to be present in a line referred to as ‘N05’, and when expressed in an *E*. *coli-*based *in vitro* system were to shown to lack activity^29^.

A possible exception to the suggestion that the splicing variants observed yield nonfunctional proteins is the species of transcript resulting from the alternative splicing of the intron in a manner that eliminates an extra nine nt at the 3′ splice junction (Fig. 6C). A transcript processed in this manner could potentially yield a product that only differs from wild type through the substitution of an Arg residue with a Gln at position 174 followed by the deletion of the next three amino acids. It would be of interest in future investigations to determine the degree by which the activity or substrate specificity may differ in a UGT76G1 variant produced in this manner.

## Methods

### Plant material and general tissue culture conditions

*Stevia rebaudiana* (Bertoni) cultigens Native 1 (NTV1) and Seed Saver 32 (SDSV32) were obtained from the Department of Horticultural Science at North Carolina State University, Raleigh, USA. Both NTV1 and SDSV32 were used for *UGT76G1* cDNA isolation and characterization studies. NTV1 was used for plant transformation.

For establishing the growth under tissue culture conditions, young shoots were collected from mature greenhouse grown plants and rinsed extensively under running tap water. Afterwards, the majority of the leaves were stripped and the segments with 2–3 nodes were further processed by an initial sterilization with 70% ethanol for 20–30 sec, followed by a 10% (v/v) bleach with 0.1% Tween 20 treatment for 10 min with agitation, and a final wash with sterile water for 3–5 times.

Clonal propagation of the NTV1 cultigen in culture included excision of 1–2 cm of the shoot apex followed by sub-cultured on ½ MS basic culture maintenance medium (Table 1). Plant cultures were maintained in a lighted incubator (16/8 hours, light/dark; 23–25 °C).

### *UGT76G1* cDNA isolation and generation of plant transformation constructs

To investigate the diversity of *UGT76G1* transcripts within Stevia, total RNA was extracted from leaves of NTV1 and SDSV32 plants using the Quick-RNA MiniPrep kit (Zymo Research, Irvine, CA) with a column purification that included DNase treatment. cDNA was synthesized using the ImProm-II Reverse Transcription System (Promega, Madison, WI). First-strand cDNAs were used as the template to carry out PCR reactions using Expand High Fidelity PCR System (Roche Applied Science, Germany) with primers specific to *UGT76G1* (Sr76G1-F1 and Sr76G1-R1, Supplementary Table 1) to amplify the region encompassing the entire open reading frame. Genomic DNA contamination was tested by using the control reactions where the reverse transcriptase enzyme was omitted. The PCR products were directly cloned into a pGEM-T Easy TA cloning vector (Promega) and transformed into *E*. *coli* competent cells. Individual colonies were randomly selected to confirm the presence of an insert, then subjected to DNA sequence analysis. cDNA sequences were aligned using MEGA5^30^ or Multalin^31^.

A cDNA clone isolated from SDSV32 which shared 100% sequence identity to accession AY345974 was cloned into the binary vector PC-GW series^32^, with hygromycin B as the selection agent (PC-GW-76G1-AY-hptII). The *UGT76G1* cDNA was placed under the transcriptional control of the constitution Cauliflower Mosaic Virus (CaMV) 35S promoter (Fig. 4). The fidelity of the transformation construct was confirmed by DNA sequencing and was introduced into *A*. *tumefaciens* EHA105 by electroporation.

### UGT76G1 *in vitro* enzymatic detection

Total RNA was extracted from *S*. *rebaudiana* cultigen SDSV leaf tissue using a PureLink RNA Mini Kit (ThermoFischer Scientific, Waltham, MA). Subsequently, cDNA was synthesized using a SuperScript II Reverse Transcriptase (ThermoFisher Scientific, Waltham, MA) using primer sequences specific to the *AY345974* and *GQ259127*-like *UGT76G1* glucosyltransferase isoforms. The PCR products were cloned into a pGEM-T Easy vector (Promega, Madison, WI) for sequence verification prior to subcloning into a Novagen pET-30 (MilliporeSigma, Burlington, MA) expression vector. Validated vectors were transformed into Novagen Rosetta gami 2(DE3) pLys S (MilliporeSigma, Burlington, MA) *E*. *coli* competent cells.

Fifty mL cultures of each construct were grown in LB medium containing 50 µg/mL kanamycin at 37 °C for 3 hours to an OD_600_ of 0.6, at which point they were induced with IPTG (to a final concentration of 1 mM) and incubated at 22 °C. Overnight cultures were centrifuged at 5,000 × *g* at 4 °C for 15 min, generating a pellet which was resuspended in lysis buffer (50 mM NaH_2_PO_4_, 300 mM NaCl, pH8), then frozen at -20 °C, thawed, then incubated on ice with 1 mg/mL of T4 lysozyme, followed by sonication on ice with six 10-second pulses. The lysate was centrifuged at 10,000 × *g* at 4 °C for 20 min, separating soluble (supernatant) from insoluble proteins (pellet resuspended in lysis buffer). Protein extracts were column-purified (HisPur Cobalt Spin Columns, ThermoFisher Scientific, Waltham, MA) and visualized by SDS-PAGE. Protein quantification was done with a Coomassie dye-based assay (G-Biosciences, St. Louis, MO). Enzyme activity assays were conducted in 50 µL of reaction buffer (50 mM K_2_HPO_4_, 3 mM MgCl_2_, pH 7.2) to which UDP-glucose (MilliporeSigma, Burlington, MA) was added as the sugar donor to concentration of 1 mM, and spiked with 2.25 µM UDP-[^14^C] glucose, 0.02µCi (PerkinElmer, Waltham, MA). The various SG substrates were added at concentrations of 50, 100, 200 and 400 µM, together with 400 ng recombinant protein (total soluble lysate or His-tag purified 76G1-AY or 76G1-GQ). The tested acceptors included stevioside, steviol-1,2-bioside (MilliporeSigma, Burlington, MA), steviol, dulcoside A, rubusoside, and rebaudiosides A, B, C, D and F (Chromadex, Irvine, CA). An empty-vector lysate was included as a control. Reactions were incubated for 2 hours at 30 °C in a thermocycler and stopped by addition of 50 µL of water-saturated butanol, which served to extract the reaction products. All reactions were run in triplicate. Ten µL aliquots of the butanol layer were spotted on 20 ×20 cm TLC plates (Silica gel 60 F_254_, MilliporeSigma, Burlington, MA) and placed in chromatography chambers containing a chloroform:methanol:water (15:10:2) solvent. Plates containing the radiolabeled SG products were read on a System 200 Imaging Scanner (Bioscan, Washington, DC) and the reaction products and standards were quantified with the WinScan software. Apparent K_m_ values were determined; it was not possible to establish V_max_ values due to the inability to reach saturating concentrations of substrate.

### Agrobacterium-mediated transformation using axillary shoot as the explant

*A*. *tumefaciens* EHA105 containing the PC-GW-76G1-AY-hptII construct was inoculated into 20 ml of YEP (Yeast extract 10 g/L, Bacto peptone 10 g/L and NaCl 5 g/L, pH 7.0) medium with 50 mg/L kanamycin and 25 mg/L rifampicin in a sterile flask. The inoculation was cultured at 28 °C in a shaker at 200 rpm for 16–20 hours. The Agrobacterium culture was centrifuged (Eppendorf, 5810 R) at 4,000 rpm for 25 min at 20 °C. The resulting pellet was suspended in Agrobacterium Inoculation medium (AgI, Table 1) and adjusted to a final OD_600_ of about 0.3. Acetosyringone was added to the suspended culture to a final concentration of 100 µM.

The explants used in this approach were dark grown axillary shoots. Explant preparation involved taking the nodal regions without leaves from tissue cultured NTV1 plants and placing them erect into Axillary Shoot Induction medium (ASI, Table 1). The nodes were cultured in the dark in an incubator (23–25 °C) for two weeks, after which the newly induced 1–3 cm long, pale green axillary shoots were harvested with a sharp surgical blade directly into the prepared Agrobacterium culture suspension as above. The axillary shoots were gently mashed with a round-end glass rod. The macerated shoots in the culture suspension medium were incubated at room temperature with gentle rocking for 60 min.

After pouring off the excess culture medium, the gently crushed shoot tissues were separated and placed on Agrobacterium-explant Co-Culture medium (CC, Table 1). The plates were cultured in the dark in an incubator (23–25 °C) for two days. The plant materials were subsequently cut into 3–5 mm pieces and arranged on Callus Induction and selection medium (CI, Table 1) with Ticar (to inhibit Agrobacterium growth) and 6 mg/L hygromycin B (Hyg, plant selection agent). The materials were again cultured in the dark in an incubator at 23–25 °C for callus induction/selection. The plant materials were subsequently sub-cultured onto fresh CI medium every 3–4 weeks.

The Hyg-resistant callus was transferred onto Differentiation Medium (DM, Table 1) with 4 mg/L Hyg. These plates were cultured in the dark or in a very low light incubator for the first 12–16 days, and then transferred to an illuminated incubator (16/8 hours, light/dark; 23–25 °C, Percival Scientific, Iowa, USA). The callus and differentiated shoot primordia were sub-cultured onto fresh DM medium every 2–3 weeks. To induce root formation, the differentiated shoots 1 cm or longer were excised with a scalpel and placed directly onto Rooting Medium (RMH, Table 1) containing 4 mg/L Hyg.

### Molecular analyses

#### PCR screening

To screen and verify the presence of the transgene in Hyg resistant plants, PCR was performed on genomic DNA isolated from rooted plants. Leaf samples (approx. 2 mm × 2 mm) were harvested from established individual plants and DNA was isolated using Shorty buffer (0.2 M Tris-HCl, pH 9.0, 0.4 M LiCl, 25 mM EDTA and 1% SDS, 10.1101/pdb.rec11660, Cold Spring Harbor Protocol 2009). The primers used for genotyping included one specific for the CaMV 35 S promoter (PC-GW-35S-F2, Supplementary Table 1) and the other corresponding to the downstream gene of interest (Sr76G1-R4, Supplementary Table 1).

#### Gene expression analysis

To investigate the relative expression levels of the *UGT76G1*, total RNA was isolated from one leaf at the second node from both transgenic and control plants grown under tissue culture conditions, using the Quick-RNA MiniPrep Kit (Zymo Research) with a column purification that included DNase treatment. First strand cDNA was synthesized using the ImProm-II Reverse Transcription System (Promega). RT-qPCR of transgene expression was measured using the SYBR Green method (iTaq Universal SYBR Green Supermix, Bio-Rad, Hercules, CA) with the Mx3005P qPCR System (Agilent Technologies, Santa Clara, CA). The RT-qPCR primers were designed to target the conserved region of both *UGT76G1* versions AY345974 and GQ259127 (SrUGT76-11/23-qF1 and -qR1, Supplementary Table 1). *UGT76G1* expression was normalized to the expression of Stevia *Actin-1* (EU751292.1, with primers SrActin-F2 and -R2, Supplementary Table 1).

#### Southern blot analysis

To validate and characterize the transgenic events, Southern blot analysis was conducted using genomic DNAs isolated from the leaves of transgenic and non-transgenic control plants. Genomic DNAs were digested with EcoRI (New England Biolabs, Ipswich, MA) which cuts once within the T-DNA construct, but not within the region of *hptII* resistance gene used for the probe (Fig. 4). EcoRI-digested genomic DNAs of each line were electrophoresed through a 1% (w/v) agarose gel and blotted onto a positively charged nylon membrane (Amersham Hybond-N+, GE Healthcare Life Sciences, Pittsburgh, PA) as described in the DIG Application Manual for Filter Hybridization (2008 by Roche Diagnostics GmbH, Mannheim, Germany). The DIG labeled probe (specific to *hptII* gene) was synthesized using the PCR DIG Probe Synthesis Kit. All labeling, hybridization and detection were carried out in accordance to the instructions in the DIG Application Manual for Filter Hybridization (2008 by Roche Diagnostics GmbH). All labeling, hybridization and detection reagents were purchased from Sigma-Aldrich (St. Louis, MO). Hybridization was carried out at 42 °C overnight. The chemiluminescent substrate CDP-Star-ready-to-use was used for detection, according to manufacturer’s instructions (Roche Diagnostics GmbH). The signal was detected by exposing the hybridized blot to X-ray film.

### SG analysis by HPLC-MS

#### Sample collection

Eleven transgenic lines and 3 independent non-transgenic controls were selected for SG profile analysis. Leaves were collected from 6–12 clonally propagated tissue cultured plants of each selected transgenic and non-transgenic line. Harvested leaves were dried in a 60 °C oven for 3 days. The dried leaves were ground to powder using a mortar and pestle.

#### Glycoside analysis by HPLC-MS

Leaf tissue was extracted with 50% methanol at a 1:100 ratio (dry weight: volume) for 30 min in 60 °C water bath. Undiluted and 100x diluted samples of each extract were then analyzed by LC-MS/Q-TOF (Agilent G6530A) with an Eclipse Plus C18 column (3 ×100 mm; 1.8 µm, Agilent). The steviol glycosides in the samples were separated by a gradient of two mobile phases: mobile phase A was water with 0.1% formic acid and mobile phase B was acetonitrile with 0.1% formic acid. The gradient started with a 1 min hold at 2% B, followed by the increase of B% from 2 to 98 over 20 min. The MS data were collected in negative mode with extended 2 GHz dynamic range. The extracted ion peak areas of the highly abundant stevioside, Reb A, and Reb C compounds were collected from the 100x diluted sample runs, while that of the other steviol glycosides with lower abundance were measured from the undiluted samples. The quantification was calculated based on the peak area and the standard curve of each steviol glycoside.

## Supplementary information

SupplementaryInformation.
