# Supplementary material for: An Efficient Stevia rebaudiana Transformation System and In vitro Enzyme Assays Reveal Novel Insights into UGT76G1 Function
Source: Sci Rep. 2020 Feb 28;10:3773. doi: 10.1038/s41598-020-60776-y (PMC7048785; doi:10.1038/s41598-020-60776-y)
Supplement: Supplementary file 1 — SupplementaryInformation. [file 41598_2020_60776_MOESM1_ESM.pdf]

SUPPLEMENTARY INFORMATION

Establishing an Efficient *Stevia rebaudiana* Transformation System and Modifying *UGT76G1* Expression in Transgenic Stevia Plants

Qian Wu[a], Christophe La Hovary[b], Han-Yi Chen[c], Xu Li[c] Hayde Eng[a], Veronica Vallejo[d],

Rongda Qu, and Ralph E. Dewey*

Supplementary Table 1. List of primers used in this research.

Sr76G1-F1	AACGTCAGTCAAACCCAATG	Used for amplifying full length UGT76G1 cDNAs
Sr76G1-R1	CTCACATAACCAACAACCATCC	Used for amplifying full length UGT76G1 cDNAs
SrUGT76-11/23-qF1	CAGATTCATCCTCGACAACG	Used for RT-qPCR analysis of transgenic plants
SrUGT76-11/23-qR1	CAGTTCGCGTCGTAATTCG	Used for RT-qPCR analysis of transgenic plants
SrActin-F2	ACCGTGTTTCCTGGTATTGC	Used as normalization control in RT-qPCR assays
SrActin-R2	CACCCTTGGAAATCCACATC	Used as normalization control in RT-qPCR assays
PC-GW-35S-F2	AAGGTGGCTCCTACAAATGC	Used to confirm 35S:76G1 transgene in transgenic plants
Sr76G1-R4	CGTTGTCGAGGATGAATCTG	Used to confirm 35S:76G1 transgene in transgenic plants

Supplementary Table 2: Glycoside profile of NTV1 and SDSV32. Leaves samples were collected from field grown plants (Clayton, North Carolina, USA) of cultigens NTV1 and SDSV32. The content is presented in µg/mg dry weight.

	**Reb A**	**Reb B**	**Reb C**	**Reb D**	**Reb E**	**Dulcoside A**	**Steviol-1,2-bioside**	**Stevioside**
NTV1	25.53	0.67	6.94	1.02	0.60	1.32	0.44	46.68
SDSV32	91.87	1.17	10.03	3.08	0.28	0.20	0.20	18.51

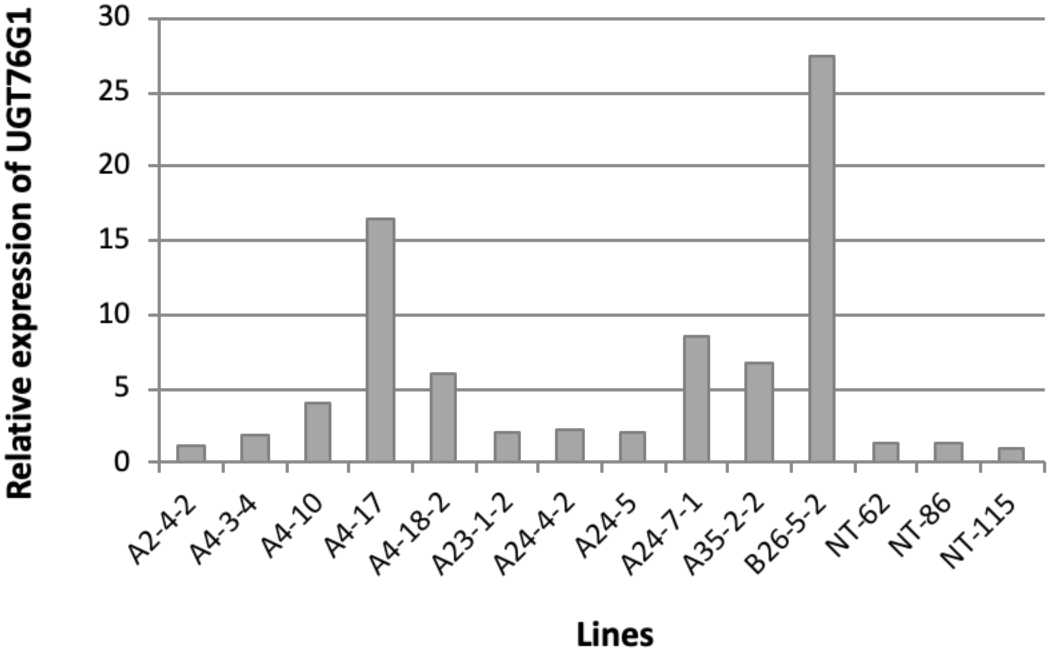

Supplementary Figure 1 RT-qPCR analysis of overexpressing *UGT76G1* transgenic Stevia plants. Total RNA was isolated from one leaf at the second internode of transgenic and control plants grown under tissue culture conditions. qRT-PCR of UGT-76G1 expression was measured using the SYBR Green method, and the primers are located in conserved regions of the four NCBI UGT-76G1 accessions. UGT-76G1 expression is represented relative to wildtype expression and normalized to Stevia *Actin-1* expression. A2, A4, A23, A24, A35 and B26 are transgenic lines, NT lines are non-transgenic.

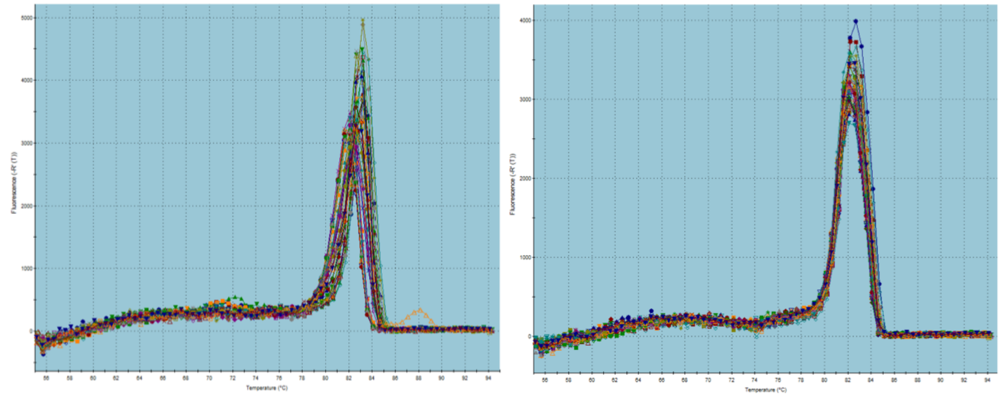

Supplementary Figure 2 Dissociation curves from the RT-qPCR amplifications of *UGT76G1* (left) and Stevia *Actin-1* (right). Samples from 11 transgenic lines and 3 non-transgenic lines were included, each sample has 3 technical replicates.
